# Non-invasive measurement of myocardial extracellular volume using T1 mapping as a novel biomarker of diffuse fibrosis in dilated cardiomyopathy

**DOI:** 10.1186/1532-429X-15-S1-E113

**Published:** 2013-01-30

**Authors:** Fabian aus dem Siepen, Sebastian A Seitz, Evangelos Giannitsis, Hugo A Katus, Henning Steen, Hassan Abdel-Aty

**Affiliations:** 1 Universitatsklinikum Heidelberg, Heidelberg, Germany

## Background

Diffuse myocardial fibrosis is frequently observed in histologies of dilated cardiomyopathy (DCM). T1-mapping techniques have the potential to measure fibrosis quantitatively and therefore provide novel cardiac magnetic resonance (CMR) insights into myocardial tissue characterization beyond the presently employed late gadolinium enhanced (LGE) techniques. Fibrosis results in expansion of the myocardial extracellular volume fraction (ECV). Recent reports indicate that T1-mapping calibrated by the blood hematocrit is a promising tool to non-invasively quantify ECV. We explored the clinical utility of this technique in a DCM cohort with different disease stages.

## Methods

**Figure 1:**
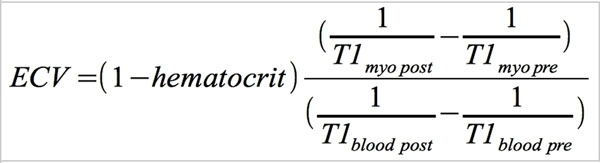


All CMR examinations were performed using 1.5 T CMR scanner (Philips Achieva). Short axis slices covering the LV were acquired using SSFP-sequences to measure volumes and ejection fraction. T1-relaxation times were measured from 62 patients (52±9 years, 39 males) with DCMP and 56 healthy volunteers (56±15 years, 37 males) before and 15 minutes after gadolinium DTPA contrast injection (Magnevist, 0.2 mmol/kg) whereas focal myocardial fibrosis was assessed in LGE images 10 minutes after i.v. injection. T1-maps were created out of 11 mid-ventricular short axis views with increasing inversion times (TI;100-4400 ms) using a single breath-hold modified Look-Locker inversion- recovery sequence (MOLLI, TR/TE=3.5/1.8 ms, flip angle=35°) in late diastole. The formula for calculating extracellular volume fractions is given in Figure 1.

## Results

**Figure 2:**
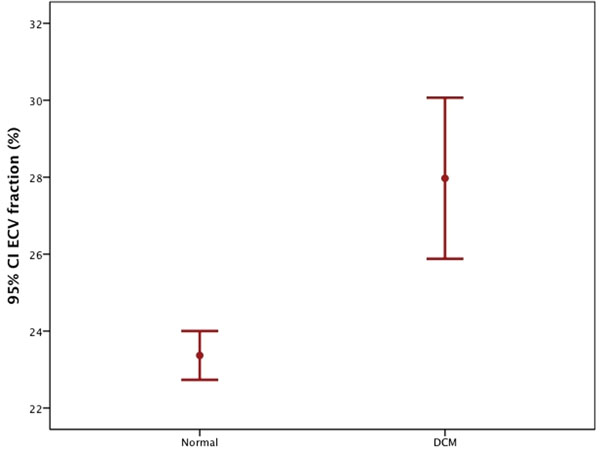


Mean LVEF was 43±12% in DCM and 62±3% in controls. Compared to controls, ECV fraction was significantly higher in DCM patients (28±8% vs 0.23±2%;p<0.0001, Figure 2). Patients with only mildly reduced LVEF had significantly larger ECV fraction than healthy controls (28±10% vs. 24±2%; p<0.03). ANOVA analysis showed significant differences between controls and patients with various DCM severity grades (p<0.02). There was no significant association between the presence of LGE and the severity of LV dysfunction. ECV was not significantly different between patients with (28±6%) or without LGE (27±9%; p=0.6). There was a weak but significant inverse correlation between ECV fraction and LVEF (r=-0.22; p=0.04) whereas no significant correlation between ECV fraction and LV end diastolic volume was found.

## Conclusions

Extracellular volume fraction as quantified by T1 mapping CMR is enlarged in DCM reflecting diffuse myocardial fibrosis, which is present even in milder disease forms. In contrast to conventional T1 mapping , ECV fraction measurement quantifies diffuse fibrosis in absolute, easy to understand units making it easier to follow up patients or to compare the amount of fibrosis in different patients' groups.

## Funding

None.

